# Impact of Exercise Intensity on Calprotectin Levels in Healthy Volunteers and Patients with Inflammatory Rheumatic Diseases

**DOI:** 10.3390/life11050377

**Published:** 2021-04-22

**Authors:** Andy Xavier, Annabelle Cesaro

**Affiliations:** I3MTO (Imagerie Multimodale Multiéchelle et Modélisation du Tissu Osseux et Articulaire)/EA 4708, Université d’Orléans, 45000 Orléans, France; andy.xavier@univ-orleans.fr

**Keywords:** calprotectin, exercise, health, chronic inflammatory rheumatic diseases

## Abstract

Exercise influences inflammatory response and immune system performance. The regular practice of a moderate activity positively regulates immunity and the inflammatory process, while intensive training depresses it and enhances inflammatory marker secretion. Calprotectin is involved in the inflammatory process, promoting neutrophil recruitment, cell degranulation, and inflammatory mediators. Furthermore, calprotectin has been associated with various inflammatory diseases, including inflammatory rheumatic diseases. The present review explores the effect of exercise on calprotectin levels in both healthy and inflammatory rheumatic conditions. Data show that the intensity duration and the type of exercise modulate calprotectin levels and participant inflammatory status. The exact role of calprotectin in the exercise response is yet unknown. Calprotectin could constitute an interesting biomarker for monitoring both the effect of exercise on the inflammatory process in healthy volunteers and the efficiency of exercise treatment programs in a patient with inflammatory rheumatic disease.

## 1. Introduction

The effect of physical exercise on the immune system and inflammatory response has been studied for over 100 years [1]. In 1902, Larrabee had already observed a marked leukocytosis following violent exercise, including many polymorphonuclear neutrophils. It is now admitted that physical exercise is a significant stress for the body, threatening its homeostasis. Exercise acts on the immune system and notably on the total number and composition of circulating leukocytes. Lymphocytes and neutrophils are the cells that are the most mobilized during exercise [2,3,4]. During acute practice, a transient increase in leukocytes has been observed. This increase returns to normal within 6 to 24 h after exercise.

Furthermore, regular exercise can have different effects depending on its intensity. Excessive training, defined as a high volume of training over a long period, leads to dysfunction of the immune system, accompanied by an increase in inflammatory mediator concentrations [5]. In contrast, regular training of moderate intensity has beneficial effects on immune systems, such as an increase in serum immunoglobulins and several other small changes in circulating numbers of immune system variables [6]. In addition to the change in counts, cell activities can be altered, such as phagocytosis, respiratory burst, and secretion of inflammatory markers [6].

Proteins S100A8, S100A9, and the heterodimer S100A8/A9, also called calprotectin, are part of the S100 calcium-binding protein family [7]. These molecules are constitutively expressed in neutrophils and monocytes/macrophages [8,9] but also in bone cells (pre-osteogenic cells, osteoblasts, and osteoclasts) and cartilage cells (chondrocytes) [10]. Under inflammatory conditions, endothelial and synovial cells also express them [10]. An expression of S100A8/A9 in muscle cells was reported by Mortensen et al. [11]. These proteins are also found in inflammatory diseases [8,10,12]. Several studies highlighted the role of calprotectin and S100A9 in rheumatoid arthritis (RA) [13,14,15,16] and showed that calprotectin could be a promising biomarker for disease activity [17,18,19,20]. Recently, various studies suggested a role for calprotectin in several other chronic inflammatory rheumatic disorders, such as juvenile chronic arthritis [21] or spondyloarthritis and ankylosing spondylitis [22,23]. Calprotectin, S100A8, and S100A9 serve as pro-inflammatory cytokines, possess innate immunity functions such as cell adhesion and chemotaxis, and show antimicrobial properties [13,24,25]. They are also involved in bone remodeling and, more particularly, in bone erosion [26,27]. Under certain circumstances, these proteins have also been shown to have anti-inflammatory roles due to their antioxidant capacity [28,29].

Herein, we discuss the effect of exercise intensity on calprotectin levels in healthy volunteers and in patients with inflammatory rheumatic diseases.

## 2. Calprotectin, a S100 Protein

### 2.1. Generalities

In the 1960s, Moore observed for the first time the S100 proteins [30]. In the 1990s, expression of S100A8 and S100A9, also called myeloid-related proteins 8 and 14 (MRP8 and MRP14), respectively, were identified in myeloid lineage cells [31,32]. They are also known as calgranulin A and B due to their calcium-binding properties. The S100A8/S100A9 heterodimer is also called calprotectin. As already mentioned, these molecules are expressed by immune cells [8,9,32], bone cells, cartilage cells [10] and are reported, under inflammatory conditions, in endothelial and synovial cells [10], and during exercise in muscle cells [11].

As mentioned above, S100A8 and S100A9 proteins can form homodimers and heterodimers. Each of these forms has been reported in the literature to exert biological functions either intracellularly or extracellularly. We know that the three forms are not always co-expressed and that their secretion can occur independently during inflammatory responses or processes [32,33]. When these molecules are in the extracellular space, they act as a warning/danger signal. Therefore, these molecules belong to the damage associated molecular pattern (DAMPs) family and are also called alarmins [34]. The following sections focus on the extracellular functions of these proteins in inflammatory situations and bone modifications.

### 2.2. Extracellular Role of the Calprotectin, S100A8, and S100A9

In this paragraph, we briefly present the extracellular roles of the calprotectin, S100A8, and S100A9. It is common for the roles reported for these proteins to be for the homodimer forms and that calprotectin was used rather as a biomarker for inflammatory disease. In the literature, studies report that murine S100A8 and human S100A8 have a chemotactic capacity for neutrophils [9,35]. In addition to their chemotactic ability, human S100A8 and human S100A9 stimulate neutrophil adhesion to fibrinogen, allowing for cell migration [9,13,24,25]. It has also been reported that S100A9 stimulates neutrophil phagocytosis [36], degranulation [37], phagocyte migration [13,38], and that it promotes the differentiation of the acute myeloid leukemia cell [39]. Blocking S100A9 in the arthritis-induced model leads to decreased disease activity by increasing cell infiltration and bone erosion [13]. In 2015, Dapunt et al. showed that S100A9 directly promotes osteoclast differentiation [40]. S100A8 also inhibits the differentiation of acute myeloid leukemia cells into mature neutrophils and monocytes [39,41] and enhances osteoclastic bone resorption in murine antigen-induced arthritis [42].

Regarding cytokine secretion, studies report that calprotectin, S100A8, and S100A9 induce pro-inflammatory cytokine secretion. S100A9 induces the secretion of pro-inflammatory cytokines such as interleukin (IL)-6, IL-8, monocyte chemoattractant protein-1 (MCP1), tumor necrosis factor-α (TNF-α), and IL-1β [13,25,43]. S100A8 induces secretion of IL-1β and TNF-α [43,44], while calprotectin induces TNF-α and IL-6 [45,46], as well as IL-8 secretion [47].

However, the roles of calprotectin, S100A8, and S100A9 are not only confined to pro-inflammatory effects [28,29]. Calprotectin appears to have the ability to trap cytokines [48,49], highlighting an anti-inflammatory capacity. Glucocorticoids and IL-10 can induce S100A8 expression by macrophages. Interestingly, S100A8 can also induce IL-10 expression [48,50,51]. S100A8 is easily oxidized, making it a good scavenger of reactive oxygen species (ROS), leading against oxidative damage [28,52,53,54]. The oxidized form of S100A8 exhibits an anti-inflammatory function [55], resulting in the reduction of mast cell degranulation and the repression of pro-inflammatory cytokine secretion [56]. Recently, a study reported the viability of S100A8 knockout mice for the first time. These mice have increased numbers of granulocyte and monocyte progenitors. They develop a severe arthritis following chemical induction, with worse clinical scores than wild-type mice, along with an increase in bone destruction [57]. The increase in bone destruction is due to the enhanced osteoclastic activity. These observations are consistent with studies suggesting an anti-inflammatory role for S100A8.

To conclude, calprotectin, S100A8, and S100A9 harbor complex and conflicting roles. These proteins can be pro-inflammatory due to exaggerated expression, resulting in an ambient inflammatory environment. On the contrary, by modulating and restoring homeostasis during inflammatory episodes, anti-inflammatory effects are observed.

## 3. Effect of Exercise Intensity on Calprotectin Level

### 3.1. Intense Physical Effort in Healthy Volunteers

As mentioned in the introduction, physical activity can influence the immune system. Regular physical activity with appropriate rest periods is beneficial for immunity, but acute bouts of intense activity can lead to opposite effects [58,59,60]. In the literature, acute exercise influences the inflammatory response, and intense endurance exercise mimics a response such as septicemia [61]. A strong mobilization of leukocytes is observed in these cases, increasing circulating inflammatory mediators [62,63,64,65]. Here we focus on the presentation of the effect of physical exercise on calprotectin.

In 2005, Fagerhol et al. studied the effect of long-distance running on plasma calprotectin levels [66]. The study involved long-distance runners: marathon (42 km), half marathon (24 km), cross country participants (30 km), and cadets participating in a ranger training race (8 days). The authors measured the plasma level of calprotectin in participants before and after the exercise, except for the cross-country group. In this case, analyses were done immediately, or at 6 and 18 h after the physical effort. For the cadets, analyses were done immediately and at 2, 4, and 8 days during the races and following 1 or 3 days of rest. The results obtained showed an increase in the plasma concentration of calprotectin in all the experimental groups, with different increase rates depending on the effort intensity. Concentration of plasma calprotectin increased by 96-, 13-, and 20-fold for the marathon, half marathon, and cross-country runners, respectively. A 7-fold increase was observed following the ranger training race, but the return to normal levels was longer than in other exercises. Prolonged physical exercise together with external factors such as stress, food, or sleep deprivation were hypothesized to cause lower decrease in calprotectin plasma concentration. Mooren et al., in 2006, obtained similar results for marathon runners [67]. In this study, blood samples were taken before and immediately after the marathon and then at 1, 3, and 24 h post-exercise. The results show an increase in calprotectin following the marathon, with a maximum level just after the end of the physical effort. The authors also observed that calprotectin concentration decreased after the first hour post-exercise and returned to regular 24 h post-exercise.

Interestingly, Mooren et al. divided the runners into two groups according to their level of training: well-trained and less well-trained [67]. An increase of plasma calprotectin concentration was observed in both groups but differed according to the training level. The well-trained group showed a higher increase in calprotectin levels than the less well-trained group. This study also showed that during a marathon, the increase in plasma calprotectin level was associated with increased leukocyte count, which unlike calprotectin does not differ according to the training status. Niemela et al. recently conducted a study of marathon runners, half-marathon runners, and cross-country skiers (24 h races) [68]. The marathon runners and skiers were endurance-trained fitness enthusiasts, whereas the half marathon runners were recreational runners. The protocol was described in the article and referred to two other publications made by the same team and the same type of participants [69,70]. Results were in line with those obtained in the two studies above mentioned and confirmed an increase in serum calprotectin concentration following long and intense efforts, as well as confirming increases in IL-8, IL-6, and IL-10 levels. This increase in cytokine levels could be associated with the regulation of tissues damages [71]. It is interesting to note that cross-country skiers with a 24 h event showed the highest calprotectin levels.

Both Niemema et al. and Mooren et al. showed an increase in the number of leukocytes following exercise. This increase coincided with that of calprotectin. Therefore, the authors hypothesized that calprotectin could serve as a marker of myeloid reactions when analyzing the immune/inflammatory response to exercise.

Finally, for the intense exercises, in 2021 Tominaga et al. investigated the changes in urinary markers of inflammation, including calprotectin, during a 3000 m time trial [72]. Ten runners carried out a 3000 m trial at an athletic field in the evening (4:00 p.m. to 6:00 p.m.) after a selected warm up. The results showed an increase in urinary calprotectin level post-exercise. The authors did not provide an explanation or hypothesis for this result.

Peake et al. in 2008, in a prolonged but moderate-intensity exercise protocol performed on an ergo cycle at 60% of maximal oxygen uptake (VO_2_max), observed an increase in calprotectin concentration of about 2.5-fold [73]. In this study, the authors also looked at the effect of temperature by putting the subjects in 18 °C immersion [73]. This change of temperature from 32 °C to 18 °C did not impact the observed increase of calprotectin. Similarly, Kim et al. studied cyclists performing a 60 min exercise at 65% of VO_2_ max at 5 °C or 21 °C and showed an increase in serum calprotectin concentration post-exercise in both cases without significant difference between the two groups [74]. The authors hypothesized that this increase observed with the number of neutrophils indicated an increase in post-exercise neutrophil activity. Interestingly, a difference between subjects acclimatized to cold before exercise who showed a significant increase in calprotectin concentration as a direct response to cold and non-acclimatized subjects was observed.

Different studies have shown an increase in plasma calprotectin concentration during short but intense exercise [66,67,75]. Fagerhol et al. showed that there is a 3-fold increase in calprotectin concentration in subjects undergoing a treadmill test, compared with the baseline value [66]. Briefly, the test was performed on an electric treadmill with an inclination of 3 °C or 6 °C. Each participant had a warm-up period at 50% of VO_2_ max, then speed increased by 1 km.t^−1^ every 30 s until the VO_2_ max peak was reached. Mooren et al., in their study, also observed an increase in calprotectin concentration after treadmill running [67]. After running at 80% of VO_2_ max and until exhaustion, results demonstrated a tripling of serum calprotectin concentration after exercise. This study also observed a post-exercise increase in two experimental groups performing concentric or eccentric efforts whose intensity corresponded to 80% of VO_2_ max. This increase was observed just after the physical effort. At 24 h after the effort, calprotectin plasma concentration returned to normal for the concentric group, contrary to results in the eccentric group

Finally, regarding short but intense exercises, in acute high-intensity interval exercise (HIE) on an ergo cycle (10 min warm-up at 50 Watts (W), immediately followed by 10 high-intensity intervals of cycling for the 60 s at 90% Wmax with 2 min of active recovery interval exercise without resistance between each high-intensity session), Fico et al. (2017), observed a doubling of the concentration of calprotectin post-exercise associated with an increase of myeloperoxidase (MPO) and MCP1 [75]. It is interesting to note that this study also compared results obtained with a continuous moderate-intensity exercise (10 min warm-up followed by 28 min of continuous exercise at 60% Wmax). Authors observed a higher increase in serum calprotectin concentration in the continuous exercise than in the interval exercise (about 5-fold and 2-fold, respectively). The authors hypothesized that acute HIE could potentially reduce the expression of inflammatory markers compared with continuous exercise.

On the other hand, Moreen et al. did not observe an immediate increase after exercise but found a 2-fold increase of circulating calprotectin after one hour of rest following treadmill running exercise at an intensity of 60% of VO_2_ max [67]. However, this increase was smaller than in the case of intense exercise, where the plasma calprotectin increase was immediate to the effort. During a short but intense exercise, Maharaj et al. showed a 3-fold increase in calprotectin concentration and an increase in IL-6, MCP1, and MPO following a 30 min treadmill run at 75% of VO_2_ max [76]. This result also underlined a significant relationship between IL-6 and calprotectin before and after exercise. This study concluded that acute aerobic exercises mediated calprotectin in healthy volunteers and was associated with the secretion of other inflammatory factors, such IL-6 and MPO.

In 2020, Goh et al. were interested in the alarmin concentration, such as calprotectin, during concurrent high intensity aerobic and resistance training [77]. In this study, volunteers followed a 3-week protocol with 3 consecutive days of training per week, with running as a warm-up, followed by bench press, squat, and shoulder row. Each week, the speed of the treadmill increased, and the number of repetitions cycles increased. Circulating calprotectin increased post-exercise by 5.8-fold, 2.5-fold, and 4.3-fold at weeks 1, 2, and 3, respectively. At week 3, the results also showed a 3.7-fold increase at 30 min post-exercise, which was not observed in weeks 1 and 2.

In parallel, one study investigated the secretion of calprotectin by the muscle in response to exercise [11]. The authors assumed that IL-6 secreted by the muscle during exercise could lead to the secretion of other factors. Using a microarray analysis and reverse transcription-polymerase chain reaction (RT-PCR) confirmation, they observed that following a recombinant human IL-6 infusion (for 3 h at a rate of 5 µg·h^−^^1^ in a volume of 25 mL·h^−1^), S100A8 and S100A9 mRNA were overexpressed by 3-fold in muscle biopsies (quadriceps), but plasma concentration was not affected. As muscle secretes IL-6 following exercise, the authors analyzed the mRNA levels of S100A8 and S100A9 in skeletal muscle in response to exercise. For this, healthy volunteers performed 3 h of cycling at approximately 60% of VO_2_ max. Quadriceps biopsies were taken post-exercise, and the results showed an approximately 5-fold increase in S100A8 and S100A9 mRNA expression compared with control. A 5-fold increase in plasma calprotectin concentration was also observed. Interestingly, the authors measured the calprotectin concentration in the arterial or femoral vein following two-legged knee extensor exercise. Results showed an increase in arterial plasma concentration of calprotectin, indirectly indicating that the secretion of calprotectin and the increase in plasma concentration following exercise could mainly come from the skeletal muscle. However, other studies showed an increase in the number of blood leukocytes, particularly neutrophils, which are the primary calprotectin source [15].

Altogether, the amount of circulating calprotectin increases following exercise, and levels depend on the intensity, duration, and type of physical exercise. Calprotectin expression also increases in skeletal muscle in response to exercise. It is important to note that the increase of calprotectin level in serum is associated with an increase in circulating leukocytes and the secretion of other inflammatory factors such as IL-6, MCP-1, MPO, and IL10 [68]. In these different studies, the authors put forward hypotheses on the role of calprotectin during exercise—in particular, its role in the extravasation of leukocytes [16] and in the activation of inflammatory signaling pathways, including NF-κB [78], and that this increase reflects an alteration in endothelial cells [66]. Furthermore, the calprotectin secretion in response to an acute bout of physical exercise could indicate neutrophil activation and could play a pivotal regulatory role in controlling inflammatory cascade. For IL-6, the increase observed could be explained in part by its release from muscle tissue. Interestingly, IL-6 had dual role as a pro-inflammatory and an anti-inflammatory. This increase could be associated with the regulation of the acute phase response, hematopoiesis, and tissues regeneration [71]. These changes in pro- and anti-inflammatory mediators (IL-6, IL-8, MCP-1, MPO, IL-10) following physical exercise highlight that a coordinated balance between anti- and pro-inflammatory cascades is needed to counteract exercise-induced inflammation and tissue damage. However, calprotectin’s exact role in response to exercise remains poorly understood to date, and further studies are warranted.

### 3.2. Moderate Exercise in Patients with Chronic Inflammatory Rheumatic Diseases

As already mentioned, intense physical activity provokes a robust immune system response, notably by the secretion of inflammatory mediators such as calprotectin. Regular physical activity with appropriate periods of rest can be beneficial to inflammatory responses [79,80]. This is mainly observed in people with inflammatory diseases. Herein, we focus on the effect of exercise treatment programs on calprotectin in the context of rheumatic diseases such as rheumatoid arthritis, juvenile idiopathic arthritis, and spondyloarthritis. Calprotectin as well as S100A8 and A9 play an essential role in the inflammatory process related to these diseases [13,14,15,16]. In addition, calprotectin correlates with the clinical score of these diseases [17,18,19,20,21,22,23].

Acar et al., in 2016, studied, after a low-intensity exercise, the modulation of calprotectin plasma concentration in patients with RA [81]. In this study, patients performed one hour of exercise three times a week for eight weeks. The sessions consisted of a warming period of 10 min, followed by aerobic exercise (20 min running treadmill, 65–70% or 75–80% of the maximal heart rate, initial and advanced program, respectively), then 20 min of muscle strengthening and finally 10 min of relaxation periods. Blood samples were taken after a period of 12 to 16 h without food intake. The results showed (1) higher calprotectin levels in the RA group and (2) a decrease in serum calprotectin concentration in RA patients after exercise not observed in the control group. This decrease was correlated with a decrease in nitric oxygen, C reactive protein, white blood cell count, and therefore with a decrease in inflammation.

Rochette et al. investigated, in two studies in patients with juvenile idiopathic arthritis, the inflammatory responses following acute exercise [82,83]. The exercise protocol used in their two studies consisted of a 20 min exercise at the beginning of the day (8:30 a.m. to 8:50 a.m.) performed on a cycle ergometer at 70% of the maximal theoretical heart rate. In the first study, blood samples were taken several times during the day (7:30 a.m., 8:30 a.m., 8:50 a.m., 9:30 a.m., 10:30 a.m., noon, 3 p.m., and 5:30 p.m.) before the day of exercise (D1), on the day of the exercise (D2), and the following day (D3) [82]. The results showed that in D1, the calprotectin and IL-6 levels were stable until noon, and then there was a peak at 3 p.m. In D2, exercise induced a 1.7-fold increase in calprotectin level immediately after exercise, associated with an increase in the pain felt. The amount of calprotectin returned to normal 3 h after exercise. This increase was not observed for IL-6, which, suggests that the increase in pain is associated with post-exercise calprotectin increase. Finally, at D3, the levels were stable for calprotectin and IL-6 except for a peak in calprotectin level at 5:30 p.m. In the second study, identical post-exercise results were obtained for calprotectin levels [83]. The authors hypothesized that exercise could be used to control calprotectin levels. This is interesting, considering that a low-dose stimulation of monocytes with calprotectin induces a state of stress tolerance, and prolonged exposure to calprotectin blocks dendritic cell differentiation and antigen presentation, resulting in a decrease of T cell response and thereby preventing amplified adaptive immune responses [84].

Levitova et al. studied the effect of an exercise treatment program on patients with spondyloarthritis [85]. The study population consisted of two subgroups of patients with ankylosing spondylitis and non-radiographic axial spondyloarthritis, two variants of spondyloarthritis. The program carried out by the patients lasted six months and was composed of 60 min sessions twice a week in physiotherapy and a home-based exercise program. The supervised programs were divided into three parts: (1) warm-up (cardiorespiratory fitness method), (2) the central part (muscles in motion, balance training abdominal muscle activity, etc.), and (3) a final part (relaxation, breathing exercise). The results obtained showed a significant decrease in the plasma concentration of calprotectin in both groups of patients. The authors also compared the concentration in a control group with two samples taken six months apart. The results showed no change in the level of calprotectin. Moreover, the authors showed a correlation between the decrease in circulating calprotectin following the program and improved symptoms. In another study, published in 2019 by Buijze et al., a slight decrease in calprotectin concentration was observed following their program but was not significant in patients with spondyloarthritis [86]. This result may be explained by the program’s content, which consisted of breathing exercise, cold exposure, and meditation without any muscular mobilization exercise, unlike Levitova et al. [85].

The studies mentioned above present decreased levels of calprotectin during long-term exercise programs in rheumatic disease patients. However, a slight increase is observed after an acute exercise, which then returns to normal level. Regular physical activity seems to lead to a reduction in the level of inflammatory molecules such as calprotectin, which also reduces the disease’s activity, as has already been demonstrated.

## 4. Conclusions

Physical exercise can be considered as a stress for the organism by modifying the immune system response, especially inflammatory response, and has been studied for over 100 years [1]. However, care must be taken when considering the effect of exercise to separate acute and regular basis exercise, as these practices do not have the same effects. Indeed, acute exercise is pro-inflammatory, while regular exercise seems to have the opposite effect [79,80]. Herein, we focus on calprotectin, a molecule involved in inflammatory process and a surrogate for inflammatory rheumatic disease activity. To date, few studies have been performed on healthy volunteers and few also on patients with inflammatory rheumatic disease.

In conclusion, intensity, duration, and type of exercise need to be considered in the different studies on calprotectin levels (substantial increase, moderate increase, or decrease). Interestingly, calprotectin expression also increases skeletal muscle response to exercise, suggesting its participation in calprotectin level increases. Some hypotheses can be proposed for the role of calprotectin during exercise, for example, a role in the extravasation of leukocytes [16], in the activation of inflammatory signaling pathways including NF-κB [78], or in reflecting an alteration in endothelial cells [66]. In patients with inflammatory rheumatic disease, a decrease in calprotectin levels during long-term exercise programs but a slight increase following an acute exercise session, followed by a return to baseline levels, was observed. These results are in line with those that show that regular physical activity leads to a reduction in the level of inflammatory molecules [79,80]. We can also hypothesize that in patients with inflammatory rheumatic diseases, this decrease in calprotectin observed after exercise programs could result in a decrease in bone erosion. Regardless, calprotectin’s exact role in exercise response in both healthy volunteers or patients with rheumatic remains poorly understood to date, and further studies will be required. However, calprotectin is an interesting biomarker for monitoring the effect of exercise on inflammatory processes in healthy volunteers and exercise treatment program efficiency in patients with inflammatory rheumatic diseases.

## Data Availability

Not applicable.

## Abbreviations

RA	rheumatoid arthritis
DAMPs	Damage Associated Molecular Pattern
MRP8	myeloid-related proteins 8
MRP14	myeloid-related proteins 14
IL	Interleukin
MCP1	Monocyte chemoattractant protein-1
TNF-α	Tumor Necrosis Factor- α
ROS	reactive oxygen species
VO_2_ max	maximal oxygen uptake
HIE	high-intensity interval exercise
W	watts
MPO	myeloperoxidase
RT-PCR	reverse transcription-polymerase chain reaction
a.m.	Ante meridiem
p.m.	Post meridiem
